# Genistein Promotes Anti-Heat Stress and Antioxidant Effects via the Coordinated Regulation of IIS, HSP, MAPK, DR, and Mitochondrial Pathways in *Caenorhabditis elegans*

**DOI:** 10.3390/antiox12010125

**Published:** 2023-01-04

**Authors:** Sai-Ya Zhang, Zi-Chen Qin, Yi-Yang Sun, Yu-Si Chen, Wen-Bo Chen, Hong-Gang Wang, Di An, Dan Sun, Yan-Qiang Liu

**Affiliations:** College of Life Sciences, Nankai University, Tianjin 300071, China

**Keywords:** genistein, anti-heat stress, antioxidant, *Caenorhabditis elegans*

## Abstract

To determine the anti-heat stress and antioxidant effects of genistein and the underlying mechanisms, lipofuscin, reactive oxygen species (ROS), and survival under stress were first detected in *Caenorhabditis elegans (C. elegans*); then the localization and quantification of the fluorescent protein was determined by detecting the fluorescently labeled protein mutant strain; in addition, the aging-related mRNAs were detected by applying real-time fluorescent quantitative PCR in *C. elegans*. The results indicate that genistein substantially extended the lifespan of *C. elegans* under oxidative stress and heat conditions; and remarkably reduced the accumulation of lipofuscin in *C. elegans* under hydrogen peroxide (H_2_O_2_) and 35 °C stress conditions; in addition, it reduced the generation of ROS caused by H_2_O_2_ and upregulated the expression of *daf-16*, *ctl-1*, *hsf-1*, *hsp-16.2*, *sip-1*, *sek-1*, *pmk-1*, and *eat-2*, whereas it downregulated the expression of *age-1* and *daf-2* in *C. elegans;* similarly, it upregulated the expression of *daf-16*, *sod-3*, *ctl-1*, *hsf-1*, *hsp-16.2*, *sip-1*, *sek-1*, *pmk-1*, *jnk-1 skn-1*, and *eat-2*, whereas it downregulated the expression of *age-1*, *daf-2*, *gst-4*, and *hsp-12.6* in *C. elegans* at 35 °C; moreover, it increased the accumulation of HSP-16.2 and SKN-1 proteins in nematodes under 35 °C and H_2_O_2_ conditions; however, it failed to prolong the survival time in the deleted mutant MQ130 nematodes under 35 °C and H_2_O_2_ conditions. These results suggest that genistein promote anti-heat stress and antioxidant effects in *C. elegans* via insulin/-insulin-like growth factor signaling (IIS), heat shock protein (HSP), mitogen-activated protein kinase (MAPK), dietary restriction (DR), and mitochondrial pathways.

## 1. Introduction

With the increase in aging population and weak environmental resistance, the incidence of age-related health disorders, including degenerative diseases, exponentially increased. The aging process is involved in many variables, including oxidative stress and thermotolerance [1,2,3,4]. The rise of anti-aging medicine puts forward a new type of health concept known as “healthy aging” [5]. Anti-oxidative and anti-heat stress may be effective methods for “healthy aging” [6,7]. Flavonoids [8], tannins, and other polyphenols [9] showed strong antioxidant activity. Genistein is a plant secondary metabolite flavonoid, widely found in legumes such as soybeans and has a variety of physiological effects, such as anti-inflammatory and antioxidant, anti-tumor [10], anti-cardiovascular disease, and anti-osteoporosis effects [11]. Because its structure is similar to that of 17β-estradiol, it can bind to estrogen receptors, it is often called a phytoestrogen, and it has a marked estradiol-related effect [12,13,14]. Genistein is produced by intestinal microbial enzymatic hydrolysis of soy products and can bind and trans-activate all three subtypes of peroxisome proliferator-activated receptors (α, δ, and γ) [15]; thus, it produces wide physiological and biochemical effects, including delaying aging and increasing life expectancy.

*C. elegans* has a short life cycle and is easy to culture; therefore, it is helpful for studying the relative issues of aging and anti-aging. Because longevity-associated signaling pathways are highly conserved, some similarity might be detected between these pathways in *C. elegans* and higher mammals, including humans [16]. It is estimated that more than 83% of the proteins in the proteome of *C. elegans* have homologs in humans [17]. Existing research shows that multiple signaling pathways, such as the mitochondrial signaling pathway, insulin/insulin-like growth factor signaling (IIS), heat shock transcription factor (HSF-1), mitogen-activated protein kinase (MAPK), dietary restriction (DR) pathways, and mitochondrial pathways, can regulate aging and anti-stress ability in *C. elegans* [17]. The IIS pathway regulates the lifespan of nematodes and mammals by regulating the conserved transcription factor DAF-16 [18]. The HSF-1 pathway prolongs lifespan by activating the expression of heat shock genes, while MAPK upregulates the longevity transcription factor (SKN-1) to combat stress-induced senescence [19,20]. Diet restriction (DR) prolongs the lifespan of *C. elegans*, depending on *eat-2* [21]. Meanwhile, CLK-1 acts in the nucleus to control the mitochondrial stress response [22]. These transcription factors are critical for stress resistance and longevity [23]. Previous studies reported that flavonoids from *Scutellariae Barbatae Herba* delay the aging of *C. elegans* through IIS [24]. A dihydroflavonoid, naringin extends the lifespan of *C. elegans* via the forkhead box (FOXO) transcription factor-16 (DAF-16) pathway [25], and proanthocyanidins alleviate β-amyloid peptide-induced toxicity by improving proteostasis through the homeostasis network regulator HSF-1 pathway in *C. elegans* [26]. In addition, genistein increasing the stress tolerance of a nematode is involved in superoxide dismutase (SOD-3) and heat shock protein (HSP-16.2) based on the experiment of transgenic strains [27]. Thus, it can be concluded that flavonoids have anti-aging effects.

Genistein is thought to have anti-stress effects and increase life expectancy; however, the underlying mechanism is still unclear. In this study, we first evaluated the effects of genistein on heat stress and oxidative stress in *C. elegans*, based on the change in ROS, lipofuscin, and survival rate. Then, we observed the nuclear localization of the aging-related transcription factor, DAF-16, and assessed the aging-related proteins, *daf-2* and HSP-16.2, and the transient expression changes of aging-related genes under heat stress and oxidative stress. It will provide mechanistic insights into the potential ability of genistein reducing stress and increasing life expectancy.

## 2. Materials and Methods

### 2.1. Materials

Peptone, agar, and hypochlorite solutions were obtained from Beijing Soleibao Technology Co., Ltd. (Beijing, China) Genistein (99%, Catalog No. 253493) was purchased from Beijing Bailingwei Technology Co., Ltd. (Beijing, China) Yeast extract, sodium hydroxide, glycerol, isopropanol, anhydrous calcium chloride, anhydrous magnesium sulfate, sodium chloride, sodium dihydrogen phosphate, potassium dihydrogen phosphate, dimethyl sulfoxide, absolute ethanol, tryptone, and chloroform were obtained from Tianjin Fengchuan Chemical Reagent Technology Co., Ltd. (Tianjin, China) Cholesterol was purchased from the Shanghai Aladdin Biochemical Technology Co., Ltd. (Shanghai, China) Levamisole hydrochloride was purchased from Nanjing Dulai Biotechnology Co., Ltd. (Nanjing, China) DNase/RNase-free water was obtained from the DEPC Department Beijing Solebold Technology Co., Ltd. (Beijing, China) RNAiso Plus and PrimeScript™ 1st Strand cDNA synthesis kits were purchased from Takara, Japan. SuperReal PreMix Plus was purchased from Beijing Tiangen Biochemical Technology Co., Ltd. (Beijing, China) Phenylmethanesulfonylfluoride (PMSF), ROS kit, and superoxide dismutase (SOD) kit were purchased from Tianjin Dingguo Technology Co., Ltd. (Tianjin, China).

### 2.2. C. elegans Strains and Maintenance

Bristol N2 (WT), EU1 [*skn-1*(zu67)], TJ375 (gpIs1 [*hsp-16.2*p::GFP]), TJ356 (zIs356 [P*daf-16*::*daf-16*a/b-gfp; rol-6]), MQ130[*clk-1*(qm30)], and LG333 [(zu135)*skn-1*::GFP] strains were provided by the Caenorhabditis Genetics Center, University of Minnesota, USA. They were maintained at 20 °C on a solid nematode growth medium (NGM) seeded with *Escherichia coli* OP50 (uracil auxotroph). OP50 (200 µL) was dropped on the center of 70 mm NGM plates, which were allowed to dry overnight and grow into a suitable colony.

### 2.3. Synchronization of C. elegans

Nematodes in the oviposition period, which contains a large number of hermaphrodite nematodes, were chosen and washed with M9 buffer. The eluent was collected and centrifuged at 400× *g* for 2 min, and the process was repeated three times. Then, lysis buffer (0.3 mL 4% sodium hypochlorite and 0.1 mL 5 M NaOH) was added to 0.6 mL sample solution, and the lysed liquid was centrifuged at 400× *g* for 2 min. The pellet was washed three times with M9 buffer and transferred to solid NGM medium for 48 h at 20 °C, and synchronous L4 (the fourth-stage larvae of nematodes) stage larvae were obtained.

### 2.4. Oxidative Stress and Heat Stress Test

The synchronized nematodes were collected and randomly divided into a blank group and an experimental group containing 200 μM genistein [the concentration was based on the result of previous studies in our laboratory [28]; and genistein was first dissolved in a little dimethyl sulfoxide, then was diluted to the required solvent with distilled water], with three parallel plates in each group, 30 per plate. After culturing for 72 h to the stage L4 at 20 °C, each group of nematodes was transferred to a new solid NGM medium for the assay of oxidative stress and heat stress. For the oxidative stress assay, 10 μL of 30% H_2_O_2_ was added to every 10 mL of NGM medium, and the number of surviving nematodes was recorded every hour until all the nematodes died [29]. For the heat stress assay, the nematodes of each group were transferred to an incubator at 35 °C for culture, and the number of surviving nematodes was recorded every hour until all the nematodes died [30]. The nematodes were considered dead when they did not respond to a gentle touch with a platinum wire on their bodies. Survival curves were drawn based on the time of nematode survival and death. These determinations were performed in three independent experiments, with each experimental group including at least 90 nematodes.

### 2.5. Determination of Lipofuscin Accumulation in C. elegans

The synchronized nematodes were collected and randomly divided into a blank group and an experimental group containing 200 μM genistein, with three parallel plates in each group, 30 per plate. Then, the fluorescence intensity of lipofuscin in *C. elegans* was observed on days number 5, 11, and 17 (from the time the eggs hatched). Then, the fluorescence intensity of lipofuscin in nematodes treated with H_2_O_2_ and 35 °C for 5 days was observed and recorded using a Leica DFC420 fluorescence microscope (excitation wavelength 340–380, emission wavelength 425 nm). The nematodes were anesthetized using levamisole hydrochloride (0.25 M) to facilitate microscopic photography. On the data processing, ImageJ software was used to open the image and extract a single channel (image–color–split channels); in the blue channel, the threshold value was adjusted, the appropriate region was selected, and the whole worm was quantified. The threshold value of the control group and genistein group of the same batch should be consistent. This experiment was performed three times, and the number of images in each experimental group was more than six.

### 2.6. Determination of ROS in C. elegans

Synchronized nematode eggs were placed on NGM plates with or without genistein. Nematodes were cultivated for 72 h to the stage L4 at 20 °C and then washed three times with M9 buffer. The nematodes were then transferred to 180 μL of M9 buffer containing 20 μL of 10 mM 2′,7′-dichlorofluorescein diacetate. The mixed liquor was incubated in the dark at 25 °C for 30 min. Then, the mixture was centrifuged at 400× *g* for 2 min. The nematode pellet was washed three times with M9 buffer and detected with a fluorescence intensity microplate reader at excitation and emission wavelengths of 485 nm and 530 nm, respectively. This experiment was performed three times with at least 500 nematodes per group.

### 2.7. Survival Determination of Mutant Strains of EU1 [skn-1(zu67)] and MQ130[clk-1(qm30)] under Oxidative and Heat Stress Conditions

Synchronized EU1 and MQ130 nematodes were collected and randomly divided into a blank group, and an experimental group with 200 μM genistein, with three parallel plates in each group, 30 per plate. After culturing for 72 h to the stage L4 at 20 °C, each group of mutant nematodes was transferred to a new solid NGM medium for the assay of oxidative stress and heat stress. For the oxidative stress assay, 30% H_2_O_2_ (10 μL) was added to every 10 mL of NGM medium (the final concentration 0.03% of H_2_O_2_), and the number of surviving EU1 and MQ130 nematodes was recorded every hour until all the nematodes died. For the heat stress assay, the EU1 nematodes and MQ130 nematodes of each group were transferred to an incubator at 35 °C for culture, and the number of surviving EU1 and MQ130 nematodes was recorded every hour until all the nematodes died. These determinations were performed in three independent experiments, with each experimental group including at least 90 nematodes.

### 2.8. Observation of the Nuclear Translocation of daf-16::GFP

The nuclear entry of DAF-16 was analyzed based on the accumulation of green fluorescence in the strain TJ356 nematodes. Synchronized nematode eggs were placed on NGM plates with or without genistein. Nematodes were cultivated to the L4 stage at 20 °C, then they were transferred to 30% H_2_O_2_ (the final concentration 0.03% of H_2_O_2_) and 35 °C for incubation for one hour. Next, the nematodes were anesthetized with levamisole hydrochloride (0.25 M), observed, and photographed under a fluorescence microscope (excitation wavelength 450–490 nm, emission wavelength 510 nm). This experiment was performed three times, and the number of shots in each experimental group was not less than six.

### 2.9. Quantification of hsp-16.2::GFP and skn-1::GFP Expression

Transgenic strains TJ375 (gpIs1 [*hsp-16.2*p::GFP]) and LG333 [(zu135)*skn-1*::GFP] were used for the analysis of *hsp-16.2*::GFP and *skn-1*::GFP expression. Synchronized nematode eggs were placed on NGM plates with or without genistein. Nematodes were cultivated to the L4 stage at 20 °C, then they were transferred to 30% H_2_O_2_ and 35 °C for incubation for one hour. Then they were anesthetized with levamisole hydrochloride (0.25 M). Finally, nematodes were observed and photographed under a fluorescence microscope (excitation wavelength 450–490 nm, emission wavelength 510 nm). On the data processing: ImageJ software was used to open the image and extract a single channel (image–color–split channels); in the green channel, the threshold value was adjusted, the appropriate region was selected, and the whole worm was quantified. The threshold value of the control group and genistein group of the same batch should be consistent. This experiment was performed three times, and the number of shots in each experimental group was not less than six.

### 2.10. Measurement of SOD Enzyme Activity

Synchronized nematodes were collected and randomly divided into blank and experimental groups with 200 μM genistein. After culturing for 72 h to the stage L4 at 20 °C, each group of mutant nematodes was ground with the lysis solution, and the activity was determined applying the SOD ELISA kit. The 560 nm absorption wavelength was used to detect the light absorption intensity using a microplate reader. This experiment was performed three times with at least 500 nematodes per group.

### 2.11. Extraction of C. elegans RNA

Synchronized nematode eggs were transferred to plates containing genistein or control plates for hatching. The hatched nematodes were grown to L4 at 20 °C and then collected by washing with deionized water. The collected nematode sample was added to 1 mL of RNAiso Plus (Takara, Kusatsu, Japan) and placed in a –80 °C refrigerator overnight. Then, the sample was slowly thawed in a 37 °C water bath, repeatedly frozen, and melted 20 times in liquid nitrogen and a 37 °C water bath, respectively. This was followed by the addition of 200 μL of chloroform, vigorous shaking for 30 s, resting at 20 °C for 10 min, and centrifugation at 3200 g for 10 min at 4 °C. The RNA containing supernatant was carefully transferred to a new vial, and an equal volume of isopropanol was added and mixed well. The mixture was incubated at 25 °C for 10 min and then centrifuged at 12,000× *g* for 10 min at 4 °C. The supernatant was carefully discarded, and 1 mL of 75% ethanol was added to the sediment, gently mixed, and centrifuged at 12,000× *g* for 5 min at 4 °C. The supernatant was discarded, and the sediment sample was air-dried for 2–5 min, followed by the addition of approximately 30 μL of DEPC water to dissolve the precipitate, and the RNA solution was stored at −80 °C. A NanoDrop 2000 spectrophotometer (Thermo, Waltham, MA, USA) was used to determine the concentration of total RNA, and RNA purity was assessed using OD260/OD280 ratios. This experiment was performed three times with at least 500 nematodes per group.

### 2.12. mRNA Analysis of Aging-Related Genes Using Quantitative Polymerase Chain Reaction (qPCR)

Using the PrimeScript™ 1st Strand cDNA Synthesis Kit, cDNA was synthesized. The reverse transcription reaction conditions were as follows: 25 °C for 10 min, 42 °C for 60 min, and 70 °C for 15 min. Quantitative PCR (qPCR) was performed using the SuperReal PreMix Plus kit. The qPCR primers used are shown in Table 1. The qPCR conditions were as follows: 95 °C for 10 min, followed by 40 cycles of 10 s at 95 °C and 30 s at 60 °C. Gene expression data were analyzed using the comparative 2^−△△Ct^ method, with *act-1* as the reference gene [23,31]. This experiment was performed in triplicates.

### 2.13. Data Treatment and Statistical Analyses

Microsoft Excel (Microsoft Corporation, Redmond, WA, USA), JMP (SAS Institute, Cary, NC, USA), GraphPad Prism 8.0 (GraphPad, La Jolla, CA, USA), and ImageJ (NIH, Bethesda, MA, USA) were used for data treatment and statistical analyses. The experimental results are presented as mean ± standard error of the mean (SEM). Significant differences between the two groups were assessed using the Student’s t-test. A log-rank test was performed for stress assays. Statistical significance was set at *p* < 0.05.

## 3. Results

### 3.1. Genistein Improves Stress Resistance of Wild-Type Nematodes Exposed to Oxidative and Heat Stress

**Figure 1:**
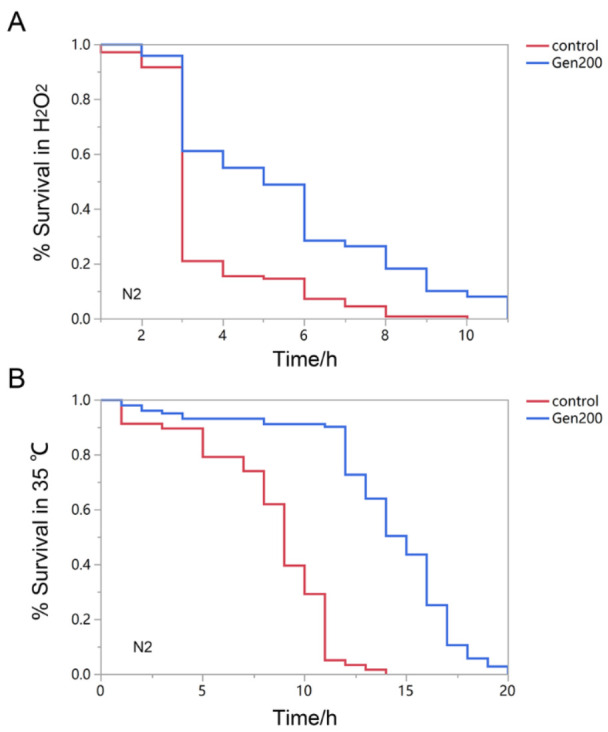
The effect of 200 μM genistein on the survival rate of *C. elegans* under stress. (**A**) The change in survival rate of *C. elegans* exposed to H_2_O_2_-induced oxidation in the genistein and control groups. (**B**) The change in survival rate of *C. elegans* exposed to heat shock at 35 °C in the genistein and control groups.

The effect of genistein on the survival rate of the H_2_O_2_- and 35 °C-treated nematodes is shown in Figure 1 and Table 2. Treatment with 200 μM genistein increased the mean survival rate of nematodes treated with H_2_O_2_ and 35 °C by 56.7% and 76.7%, respectively (*p <* 0.01).

### 3.2. Genistein Lowered Lipofuscin Accumulation in C. elegans under Control, Heat and Oxidative Conditions

**Figure 2:**
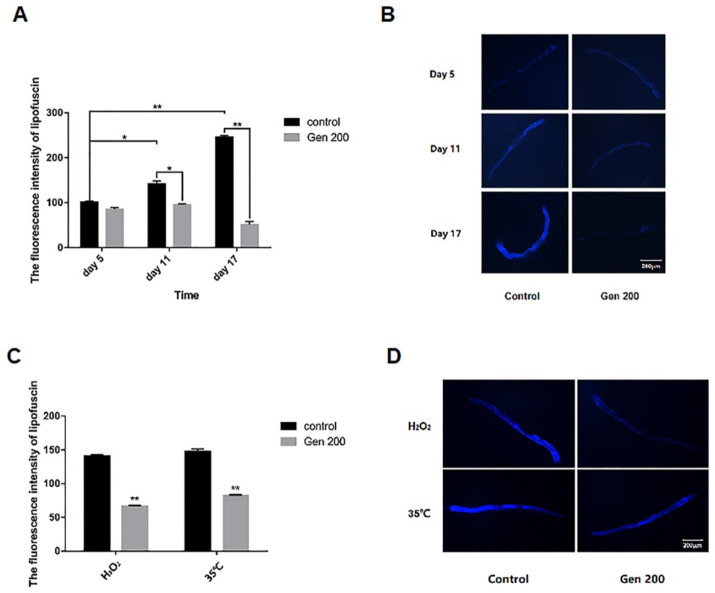
The effect of 200 μM genistein on lipofuscin accumulation in *C. elegans* under control, H_2_O_2_ and 35 °C conditions. (**A**) Relative fluorescence intensity of lipofuscin in *C. elegans* (% of control) on days 5, 7, and 11 under control conditions. (**B**) Photomicrographs of lipofuscin fluorescence in *C. elegans* on days 5, 7, and 11 under control conditions. (**C**) Relative fluorescence intensity of lipofuscin in *C. elegans* on day 5 in H_2_O_2_ and 35 °C conditions. (**D**) Photomicrograph of lipofuscin fluorescence in *C. elegans* on day 5 in H_2_O_2_ and 35 °C conditions (* p < 0.05; ** p < 0.01).

The results of lipofuscin accumulation in *C. elegans* under control, H_2_O_2_, and 35 °C conditions are shown in Figure 2. Under control conditions, the accumulation of lipofuscin increased with an increase in the number of days, and the accumulation of lipofuscin in the nematodes on the 11th and 17th day was 1.39 and 2.12 times that on the 5th day (Figure 2A,B). Genistein significantly reduced the accumulation of lipofuscin in nematodes by 32.6% (*p <* 0.05) and 79.0% (*p <* 0.01) on days 11 and 17, respectively (Figure 2A,B). In addition, on day 5, genistein reduced the accumulation of lipofuscin by 52.5% and 44.4% in *C. elegans* under heat and oxidative conditions, respectively (Figure 2C,D) (*p <* 0.01).

### 3.3. Genistein Lowered ROS Accumulation in H_2_O_2_-Treated Nematodes, and Increased the SOD Activity of Nematodes under Control, 35 °C and H_2_O_2_ Conditions

**Figure 3:**
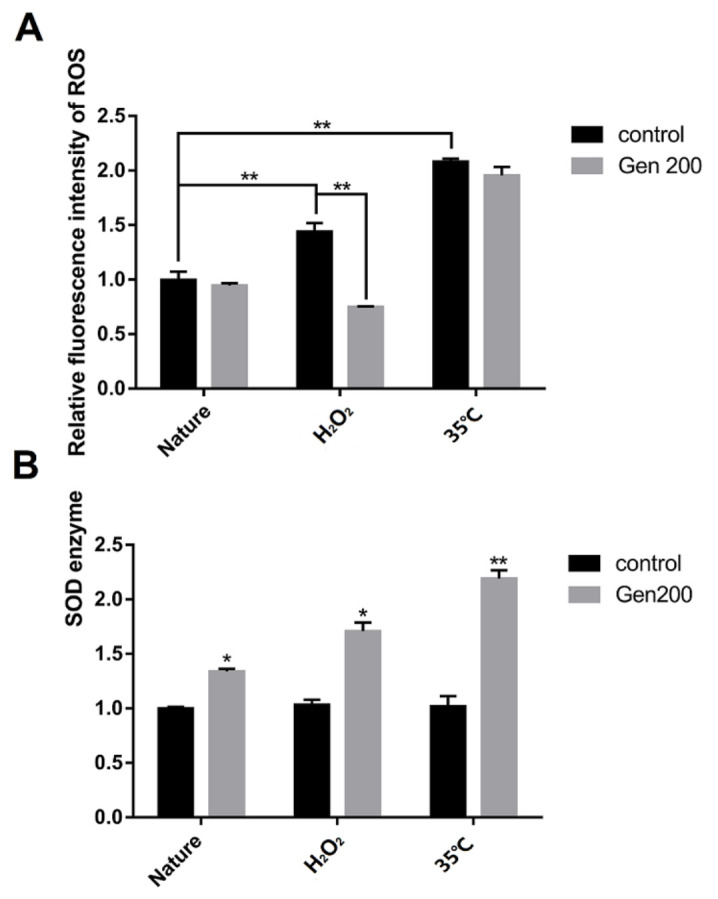
Under control, H_2_O_2_, and 35 °C conditions, 200 μM genistein influences ROS accumulation and the activity of superoxide dismutase (SOD) in the stage L4 *C. elegans*. (**A**) The effect of genistein on the accumulation of ROS in *C. elegans* under control, H_2_O_2_, and 35 °C conditions. (**B**) The effect of genistein on the activity of SOD in *C. elegans* under control, H_2_O_2_, and 35 °C conditions (* p < 0.05; ** p < 0.01).

The effect of genistein on ROS accumulation and SOD activity of the differently treated nematodes are shown in Figure 3. Genistein reduced the accumulation of ROS by 47.9% in H_2_O_2_-treated nematodes (*p <* 0.01). However, genistein produced no obvious effect in nematodes treated at 35 °C (Figure 3A). Genistein increased SOD activity by 34.1% (*p* < 0.05), 67.5% (*p <* 0.05), and 117.4% (*p* < 0.01) under control, H_2_O_2_, and 35 °C conditions, respectively (Figure 3B).

### 3.4. Genistein Partially Improved the Survival Rate of EU1 [skn-1(zu67)] Mutants, but It Did Not Significantly Change the Survival Rate of MQ130[clk-1(qm30)] Mutant under Heat and Oxidative Conditions

**Figure 4:**
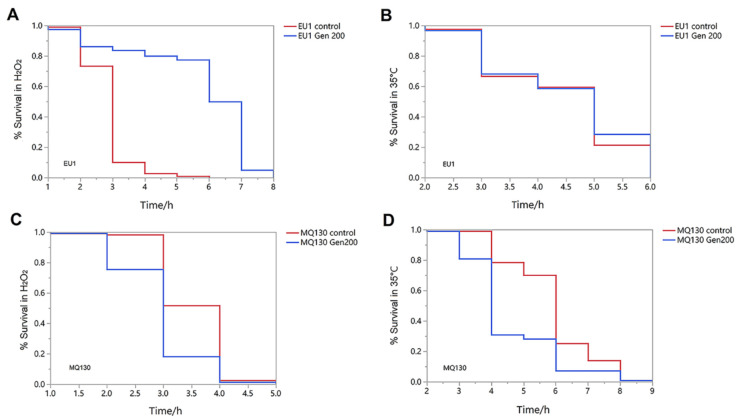
The effect of 200 μM genistein on the survival rate of EU1 and MQ130 mutant nematodes at L4 under H_2_O_2_ and 35 °C conditions. (**A**) The effect of genistein on the survival rate of EU1 mutant exposed to H_2_O_2_-induced oxidation. (**B**) The effect of genistein on the survival rate of EU1 mutant exposed to heat shock at 35 °C. (**C**) The effect of genistein on the survival rate of MQ130 mutant exposed to H_2_O_2_-induced oxidation. (**D**) The effect of genistein on the survival rate of MQ130 mutant exposed to heat shock at 35 °C.

From Figure 4, genistein increased the mean survival rate of *skn-1* (EU1) mutant strains by 93.4% under oxidative conditions (Figure 4A) (*p* < 0.01). However, genistein did not influence the survival rate curves of EU1 mutants under heat stress conditions (Figure 4B). Additionally, genistein did not significantly change the survival rate curves of the MQ130 mutants under heat and oxidative conditions (Figure 4C,D).

### 3.5. Genistein Increased the Nuclear Translocation of DAF-16 Transcription Factor under 35 °C and H_2_O_2_ Conditions

**Figure 5:**
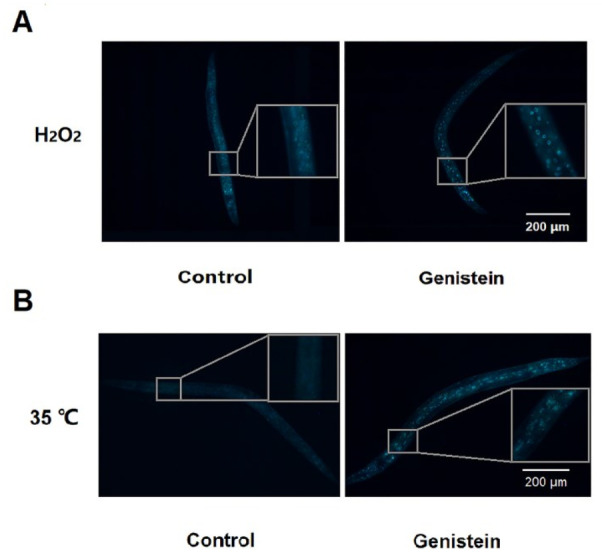
The effect of 200 μM genistein on the nuclear localization of DAF-16::GFP in the stage L4 *C. elegans* under H_2_O_2_ and 35 °C conditions for one hour. (**A**) The effect of genistein on the nuclear localization of DAF-16::GFP in *C. elegans* exposed to H_2_O_2_-induced oxidation. (**B**) The effect of genistein on the nuclear localization of DAF-16::GFP in *C. elegans* exposed to heat shock at 35 °C.

The effects of genistein on the nuclear translocation of the DAF-16 transcription factor are shown in Figure 5. From Figure 5A, the DAF-16::GFP fluorescence of the genistein-treated group under H_2_O_2_ conditions was concentrated in the nucleus, while the DAF-16::GFP fluorescence of the control group was concentrated in the cytoplasm and diffusely distributed. Moreover, under 35 °C conditions, the DAF-16::GFP fluorescence of the genistein-treated group was concentrated in the nucleus, and the DAF-16::GFP fluorescence of the control group was concentrated and dispersed in the cytoplasm (Figure 5B).

### 3.6. Genistein Enhanced the Accumulation of HSP-16.2 Protein in Nematodes under Control, 35 °C, and H_2_O_2_ Conditions

**Figure 6:**
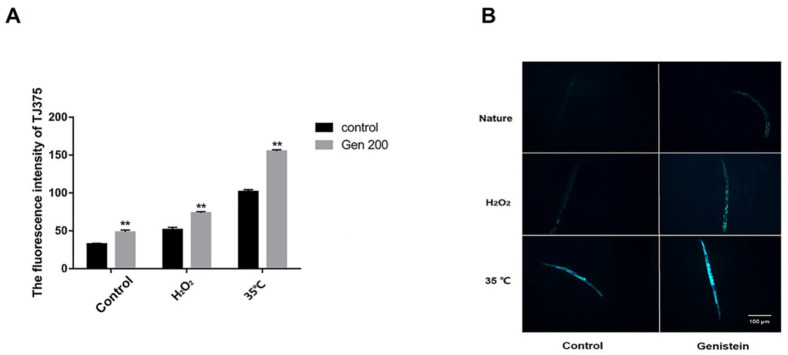
Under control, H_2_O_2_, and 35 °C conditions, 200 μM genistein influences the accumulation of HSP-16.2 protein in the stage L4 *C. elegans*. (**A**) The effect of genistein on the relative fluorescence intensity of HSP-16.2::GFP in *C. elegans*. (**B**) Micrograph of HSP-16.2::GFP fluorescence in *C. elegans* (** p < 0.01).

We assessed the accumulation of HSP-16.2 using the *C. elegans* strain TJ375 (HSP-16.2::GFP), which expresses the GFP-fused HSP-16.2 (Figure 6). From Figure 6B, under control, 35 °C, and H_2_O_2_ conditions, the green fluorescence of HSP-16.2 protein in the genistein-treated group was significantly higher than that in the control group. Based on Figure 6A, compared with the control group, the fluorescence intensity of HSP-16.2::GFP in the genistein-treated group was significantly enhanced by 41.8%, 42.5%, and 52.3% under natural, oxidative stress, and heat stress conditions, respectively (*p <* 0.01).

### 3.7. Genistein Enhanced the Accumulation of SKN-1 Protein in Nematodes under 35 °C and H_2_O_2_ Conditions

**Figure 7:**
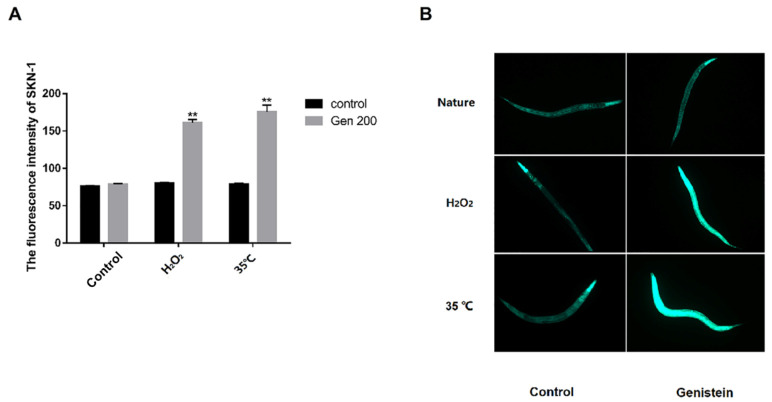
The accumulation of SKN-1 protein in the stage L4 *C. elegans* under control, H_2_O_2_, and 35 °C conditions is influenced by 200 μM genistein. (**A**) The effect of genistein on the relative fluorescence intensity of SKN-1::GFP in *C. elegans*. (**B**) Micrograph of SKN-1::GFP fluorescence in *C. elegans* (** p < 0.01).

Compared with the control group, the intensity of the green fluorescence of SKN-1 protein in the genistein-treated group did not significantly change under control conditions (Figure 7A,B). From Figure 7B, under oxidative and heat stress conditions, the green fluorescence of SKN-1 protein in the genistein-treated group was significantly higher than that of the control group, and compared with the control group, the fluorescence intensity of the genistein-treated was increased by 100.2% and 122.7%, respectively (Figure 7A) (*p <* 0.01).

### 3.8. Genistein Regulated the Expression of Messenger RNA (mRNA) in C. elegans under 35 °C and H_2_O_2_ Conditions

**Figure 8:**
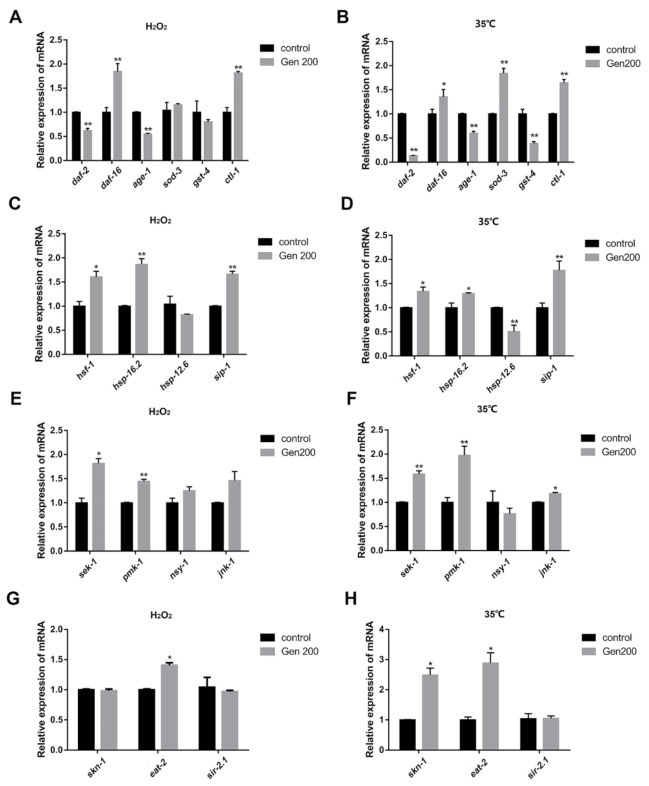
Under H_2_O_2_ and 35 °C conditions, 200 μM genistein influences the mRNA level of stress resistance genes in the stage L4 C. elegans. (**A**) The effect of genistein on the mRNA of *daf-16*, *sod-3*, *ctl-1*, *daf-2*, *age-1*, and *gst-4* in *C. elegans* under H_2_O_2_ conditions. (**B**) The effect of genistein on the mRNA of *daf-16*, *sod-3*, *ctl-1*, *daf-2*, *age-1*, and *gst-4* in *C. elegans* under 35 °C conditions. (**C**) The effect of genistein on the mRNA of *hsf-1*, *hsp-16.2*, *hsp-12.6*, and *sip-1* in *C. elegans* under H_2_O_2_ conditions. (**D**) The effect of genistein on the mRNA of *hsf-1*, *hsp-16.2*, *hsp-12.6*, and *sip-1* in *C. elegans* under 35 °C conditions. (**E**) The effect of genistein on the mRNA of *sek-1*, *pmk-1*, *nsy-1*, and *jnk-1* in *C. elegans* under H_2_O_2_ conditions. (**F**) The effect of genistein on the mRNA of *sek-1*, *pmk-1*, *nsy-1*, and *jnk-1* in *C. elegans* under 35 °C conditions. (**G**) The effect of genistein on the mRNA of *skn-1*, *eat-2*, and *sir-2.1* in *C. elegans* under H_2_O_2_ conditions. (**H**) The effect of genistein on the mRNA of *skn-1*, *eat-2*, and *sir-2.1* in *C. elegans* under 35 °C conditions (* *p* < 0.05; ** *p* < 0.01).

The results of the effect of genistein on the mRNA expression of daf-2, age-1, daf-16, sod-3, gst-4, ctl-1, hsf-1, hsp-16.2, hsp-12.6, sir-2.1, sek-1, skn-1, pmk-1, nsy-1, jnk-1, sip-1, and eat-2 in C. elegans at 35 °C and H_2_O_2_ conditions are shown in Figure 8. Under H_2_O_2_ conditions, genistein treatment upregulated the relative expression levels of daf-16, ctl-1, hsf-1, hsp-16.2, sip-1, sek-1, pmk-1, and eat-2 by 84.4%(*p <* 0.01), 81.6%(*p <* 0.01), 60.4% (*p <* 0.05), 86.6% (*p <* 0.01), 66.7% (*p <* 0.01), 81.8% (*p <* 0.05), 44.6% (*p <* 0.01), and 40.7% (*p <* 0.05), respectively, but downregulated the relative expression levels of daf-2 and age-1 by 37.8% (*p <* 0.01), and 44.9% (*p <* 0.01), respectively, and no significant effect was observed on sod-3, gst-4, hsp-12.6, nsy-1, jnk-1, skn-1, and sir-2.1 compared with the control. Similarly, genistein treatment upregulated the relative expression levels of daf-16, sod-3, ctl-1, hsf-1, hsp-16.2, sip-1, sek-1, pmk-1, jnk-1, skn-1, and eat-2 by 35.3% (*p <* 0.05), 84.1% (*p <* 0.01), 64.4% (*p <* 0.01), 33.7% (*p <* 0.05), 29.0% (*p <* 0.05), 77.7% (*p <* 0.01), 58.7% (*p <* 0.01), 97.7% (*p <* 0.01), 18.0% (*p <* 0.05), 182.3% (*p <* 0.05), and 177.2%(*p <* 0.05), but downregulated the relative expression levels of daf-2, age-1, gst-4, and hsp-12.6 by 86.4% (*p <* 0.01), 39.9% (*p <* 0.01), 60.9% (*p <* 0.01), and 49.4% (*p <* 0.01), respectively, and no significant effect was observed on nsy-1 and sir-2.1 at 35 °C compared with the control.

## 4. Discussion

Previous studies showed that genistein inhibits the aging of human umbilical vein endothelial cells [32] and attenuates the senescence of vascular smooth muscles [33]. The increase in lifespan was accompanied by anti-stress effects in *C. elegans* [34,35,36]. In this study, we evaluated whether genistein treatment improved heat stress and oxidative stress in *C. elegans*. The results show that genistein could protect the nematodes and prolong the average life and maximum life of nematodes treated with H_2_O_2_ at 35 °C. Genistein significantly decreased the accumulation of lipofuscin in *C. elegans* under H_2_O_2_ and 35 °C stress conditions. As for the quantitative determination of lipofuscin, it was pointed out in the literature that only detection of blue fluorescence changes at low levels before and after death, which may reflect the proportion of dead or nearly dead individuals in the sample; at the same time, blue fluorescence should be accepted as reliable in the current paper, and we will make more attempts including determining red autofluorescence and adding the number of worms [37,38,39,40].

The IIS pathway is an evolutionarily conserved mechanism that is involved in the longevity and metabolism of different species [41]. Several genes are involved in the IIS pathway; *daf-2* encodes a receptor tyrosine kinase homolog, and *age-1* encodes a phosphatidylinositol 3-kinase (PI3K) homolog, *daf-16* is an ortholog of human FOXO1, which encodes DAF-16, *sod-3* encodes a superoxide dismutase gene, *gst-4* encodes a glutathione transferase, and *ctl-1* encodes a catalase [18,42]. The initiation of the kinase cascade depends on the phosphorylation of the insulin/IGF-1 transmembrane receptor (IGFR) ortholog, the forkhead box (FOXO) transcription factor-2 (DAF-2), and then regulates its downstream signaling molecule, AGE-1 (phosphatidylinositol kinase) [43]. This signaling regulated the nuclear translocation of DAF-16 [44]. This further activated the expression of a large number of anti-stress and longevity genes, such as s*od-3*, *gst-4*, and *ctl-1*, which eventually led to the enhancement of the anti-aging ability of *C. elegans* [18,45]. Our results show that genistein treatment significantly increased the mRNA level of *sod-3* at 35 °C and increased the mRNA levels of *ctl-1* and SOD enzyme activity under H_2_O_2_ and 35 °C stress conditions in *C. elegans*. These results suggest that the effects of genistein, such as prolonging the lifespan and improving the anti-heat and antioxidant capacity, might result from the activation of SOD-3 and CTL-1 through the IIS pathway in *C. elegans*.

Some heat-related proteins can protect nematodes from stress [46,47]. The HSP protein family was used as a predictor of longevity in *C. elegans*, because it is closely related to heat tolerance and longevity [48,49]. The representatives of the HSP protein family were HSP-12.6, HSP-16.2,SIP-1 (the PDZ-domain containing protein), and HSF-1 [50]. HSP-12.6 is a heat shock protein located in the cytoplasm and is an ortholog of human HSPB2 (heat shock protein family B (small) member 2) [51]. HSP-16.2 is another heat shock protein located in the cytoplasm, which is an ortholog of human CRYAB (crystallin alpha B) [52]. SIP-1 is a member of the alpha-crystallin/HSP20 family and is involved in thermal defense and aging processes [53]. To be precise, these protein expressions interact with each other and are mainly regulated by HSF-1 [54], an ortholog of human HSF-2 (heat shock transcription factor 2), which protects the proteins from the damage caused by extrinsic environmental stress or intrinsic age-related deterioration in *C. elegans* [46,55,56,57]. Our results show that the resistance of nematodes and the mRNA levels of *hsp-16.2*, *sip-1*, and *hsf-1* remarkably increased after genistein treatment under H_2_O_2_ condition; and the mRNA levels of *hsp-16.2*, *sip-1*, and *hsf-1* remarkably increased after genistein treatment, while *hsp-12.6* was significantly downregulated at 35 °C. Moreover, genistein treatment significantly enhanced the expression of HSP-16.2 under control, H_2_O_2_, and 35 °C conditions; indicating that genistein, which improves resistance under H_2_O_2_ and 35 °C stress conditions, might regulate in the HSP pathway.

The JNK and p38/MAPK signaling pathways were also implicated in a variety of biological functions in *C. elegans*, including response to stress [58]. ROS reflects the level of oxidative free radicals in the cells and are involved in cell senescence and epigenetic regulation of senescence [59,60]. The above results show that genistein significantly reduced ROS production in the cells under oxidative stress, and thus improved the survival rate of nematodes under oxidative stress. Several genes are involved in the MAPK pathway. For example, *nsy-1* encodes a homolog of the human apoptosis signal-regulating kinase (ASK1, MAPKKK) and *jnk-1*, a JUN kinase, which is an ortholog of human MAPK10 and MAPK8 [61,62,63]; *sek-1*, an ortholog of human MAP2K6, has several functions, including MAPK binding activity, and *pmk-1* encodes a p38 MAPK homolog [64]. SKN-1 is the *C. elegans* ortholog of mammalian Nrf/CNC proteins and plays a role in a wide range of detoxification processes, as well as in immunity, oxidative stress defense, and metabolism [65]. Their regulatory mechanism might be mediated through *nsy-1*; it activates *jnk-1* and *sek-1*, and the latter further activates *pmk-1* [66]. Activation of the MAPK pathway leads to the activation of *skn-1*, which further regulates the expression of downstream antioxidant protein detoxification enzymes, such as the SOD enzyme [67,68]. This experiment showed that genistein elevated the expression of genes such as *sek-1*, *pmk-1*, *skn-1*, and *sod* in H_2_O_2_-treated nematodes. Similarly, genistein elevated the expression of genes such as *sek-1*, *pmk-1*, *jnk-1*, *skn-1*, *skn-1*, and *sod* at 35 °C. The interesting thing is, genistein could also increase SOD under control conditions. However, there was a mismatch between gene expression levels and enzyme activity. Studies show that the mRNA expression might not be synchronized with protein expression of the enzyme, and microRNA regulation in transcription, ubiquitination, and phosphorylation in protein activation might be an uncertain factor [69]. However, we will confirm this idea with more data or experiments. These results indicate that the effect of genistein on the survival rate of *C. elegans* under oxidative and heat stress conditions might be attributed to the MAPK pathway. On the other hand, genistein increased the survival time of the *skn-1* (EU1) mutant under oxidative conditions but did not increase survival time under heat stress conditions, which indicated that *skn-1* was essential for genistein to improve the survival of *C. elegans* under heat stress, but was not essential under oxidative stress conditions. These results indicate that the mechanism by which genistein adjusted the resistance of *C. elegans* to stress was not exactly the same under different stress conditions.

In addition, some studies showed that the DR pathway can induce longevity in *C. elegans*, and that the longevity effect depends on *eat-2* [52]; *eat-2* enabled acetylcholine-gated cation-selective channel activity and was involved in several processes in *C. elegans*, including feeding behavior. Additionally, *eat-2* is an ortholog of human CHRFAM7A and FAM7A. The results show that genistein increased the mRNA expression of *eat-2* under oxidative and heat stress conditions. These results indicate that the effects of genistein in improving the resistance of *C. elegans* might benefit from the DR pathway.

Furthermore, *sir-2.1*, enabled deacetylase activity, is an ortholog of human SIRT1 (sirtuin 1), which is involved in several processes, including determination of the adult lifespan, histone modification, and the intrinsic apoptotic signaling pathway in response to DNA damage in *C. elegans* [70]. Studies show that *sir-2.1* plays an important role in regulating the genes related to longevity in *C. elegans* [71]; *sir-2.1* and 14-3-3 act in parallel to the insulin-like pathway to activate DAF-16 and extend the lifespan [72]. However, no significant difference was observed in the mRNA expression of *sir-2.1* in *C. elegans* under H_2_O_2_ and 35 °C conditions after genistein treatment. One possible interpretation is that *sir-2.1* did not play a role in genistein-enhanced resistance in *C. elegans* under H_2_O_2_ and 35 °C conditions.

Lastly, the monooxygenase CLK-1, which is homologous to human COQ7, was previously reported to act in the nucleus to control the mitochondrial stress response and lifespan [73]. This pathway is conserved from *C. elegans* to humans, and regulates mitochondrial reactive oxygen metabolism and mitochondrial unfolded protein response [22]. We found that genistein failed to prolong the survival time of nematodes in the deletion mutant MQ130, which lacks *clk-1* under oxidative and heat stress conditions. This indicated that the mitochondrial pathway was essential for genistein-enhanced resistance in *C. elegans* under H_2_O_2_ and 35 °C conditions.

Previous data suggest that these pathways interact with each other [74]. For example, the expression of *hsp-16.2* is mainly regulated by HSF-1. However, it is also affected by the IIS pathway in *C. elegans* [75]. In addition, SKN-1, which regulates the MAPK pathway, is also an important direct target of IIS, and DAF-16 regulates its activity by reducing the nuclear localization of SKN-1 [76]. Our results suggest that genistein resistance to H_2_O_2_ and 35 °C is related to IIS, HSP, MAPK, DR, and mitochondrial signaling pathways, and is also involved in the interactions of these signaling pathways.

## 5. Conclusions

In summary, genistein improved resistance to oxidant and heat stress and increased the lifespan in *C. elegans* through the coordinated regulation of IIS, HSP, MAPK, DR, and mitochondrial signaling pathways. This points out the possible applications of genistein as an anti-heat, antioxidant, and anti-aging drug. However, more convincing data from in-depth experiments using mammalian models are required to extrapolate the biotransformation pathways of genistein in mammals, including humans.

## Figures and Tables

**Table 1:** The primer sequence of the genes.

Genes	Forward Primer	Reverse Primer
daf-16(NM_001381205.1)	AGGAGTCGAAGCCGATTAAGAC	GGTAGTGGCATTGGCTTGAAG
daf-2(NM_065249.7)	TACTTGAATCGGGCGTCGTT	GACGACTTCAACAACCGCTG
age-1(NM_064061.6)	CTCCTGAACCGACTGCCAAT	AAATGCGAGTTCGGAGAGCA
gst-4(NM_069447.8)	CCCATTTTACAAGTCGATGG	CTTCCTCTGCAGTTTTTCCA
sod-3(NM_078363.9)	TGGCTAAGGATGGTGGAGAA	GCCTTGAACCGCAATAGTGAT
ctl-1 (NM_064578.6)	CGATACCGTACTCGTGATGAT	CCAAACAGCCACCCAAATCA
skn-1(NM_171345.6)	CGTCCAACCAACCACATCATCTC	ATCTTCCAATTCGGCTTTT
sir-2.1(NM_001268555.5)	AAATCTTCCCAGGACAGTTCGTA	ATGGGCAACACGCATAGCA
eat-2(NM_064558.6)	ACCATGGGGAATTTGCAACG	GGATTTGCGTGAGGGGTATGA
hsp-16.2(NM_001392482.1)	CTGCAGAATCTCTCCATCTGAGTC	AGATTCGAAGCAACTGCACC
hsf-1(NM_060630.7)	ATGTACGGCTTCCGAAAGATGA	TCTTGCCGATTGCTTTCTCTTAA
hsp-12.6(NM_069267.4)	TGGAGTTGTCAATGTCCTCG	GACTTCAATCTCTTTTGGGAGG
sip-1(NM_066915.5)	CGAGCACGGGTTCAGCAAGAG	CAGCGTGTCCAGCAGAAGTGTG
jnk-1(NM_001026099.6)	TGGAACCAGCCAATTCCCAA	TCACAACACTCTGCTCGCAT
nsy-1(NM_001383826.1)	AGCGGCTCGATCAACAAGAA	CCCATTCCACCGATATGCGA
sek-1(NM_076921.8)	CACTGTTTGGCGACGATGAG	ATTCCGTCCACGTTGCTGAT
pmk-1(NM_068964.7)	CCAAAAATGACTCGCCGTGA	CTTTTGCAGTTGGACGACGA

**Table 2:** The effect of 200 μM genistein on the lifespan of *C. elegans* under H_2_O_2_ and 35 °C stress.

Group	Number	Mean Lifespan (h)	Median Survival (Days)	Maximum Lifespan (Days)	*p* Value
Control(H_2_O_2_)	109	3.541 ± 0.15	3	10	_
Gen (H_2_O_2_)	102	5.531 ± 0.39	5	11	<0.01
Control (35 °C)	103	8.362 ± 0.41	9	14	_
Gen (35 °C)	107	14.02 ± 0.39	15	20	<0.01

*p* < 0.01, compared with Control(H_2_O_2_) and Control (35 °C), respectively.

## Data Availability

No other data be available except the published.

## Abbreviations

CAT, catalase; C. elegans, Caenorhabditis elegans; DNA, deoxyribonucleic acid; DR, dietary restriction pathway; FOXO1, Recombinant Forkhead Box Protein O1; HSF-1, heat shock factor-1; HSPB2, Recombinant Human Heat shock protein beta-2; HSPs, heat shock proteins; IGF-1, insulin-like growth factors-1; IIS, insulin-like growth factor1 signaling pathway; JNK, Jun kinase; MAPK, the generic mitogen-activated protein kinases signaling pathway; mRNA, messenger RNA; Nrf2, NF-E2-related factor 2; ROS, reactive oxygen species; and SOD, superoxide dismutase.
